# Sex Differences in Mental Rotation and How They Add to the Understanding of Autism

**DOI:** 10.1371/journal.pone.0124628

**Published:** 2015-04-17

**Authors:** Alexandra C. Zapf, Liv A. Glindemann, Kai Vogeley, Christine M. Falter

**Affiliations:** 1 Department of Clinical and Developmental Neuropsychology, University of Groningen, Grote Kruisstraat 2/1, 9712 TS, Groningen, The Netherlands; 2 Department of Psychiatry, University Hospital Cologne, Kerpener Str. 62, 50924 Cologne, Germany; 3 Research Center Juelich, Institute of Neuroscience and Medicine, Cognitive Neuroscience (INM-3), Wilhelm-Johnen-Straße, 52425 Juelich, Germany; University of Tuebingen Medical School, GERMANY

## Abstract

The most consistent cognitive sex differences have been found in the visuo-spatial domain, using Mental Rotation (MR) tasks. Such sex differences have been suggested to bear implications on our understanding of autism spectrum disorders (ASD). However, it is still debated how the sex difference in MR performance relates to differences between individuals with ASD compared to typically developed control persons (TD). To provide a detailed exploration of sex differences in MR performance, we studied rotational (indicated by slopes) and non-rotational aspects (indicated by intercepts) of the MR task in TD individuals (total *N* = 50). Second-to-fourth digit length ratios (2D:4D) were measured to investigate the associations between prenatal testosterone and performance on MR tasks. Handedness was assessed by the use of the Edinburgh Handedness Inventory in order to examine the relation between handedness and MR performance. In addition, we investigated the relation of spatial to systemising abilities, both of which have been associated with sex differences and with ASD, employing the Intuitive Physics Test (IPT). Results showed a male advantage in rotational aspects of the MR task, which correlated with IPT results. These findings are in contrast to the MR performance of individuals with ASD who have been shown to outperform TD persons in the non-rotational aspects of the MR task. These results suggest that the differences in MR performance due to ASD are different from sex-related differences in TD persons, in other words, ASD is not a simple and continuous extension of the male cognitive profile into the psychopathological range as the extreme male brain hypothesis (EMB) of ASD would suggest.

## Introduction

Men and women are cognitively similar and different at the same time, depending on the cognitive domain studied. The ability to mentally rotate objects has been the most consistently reported and most robust sex-related difference in the cognitive domain favouring males over females (e.g.,[1, 2, 3, 4, 5]).

In a mental rotation (MR) task [6] the participant is instructed to mentally manipulate (e.g. rotate) and compare two- or three-dimensional figure to decide whether the figures are the same but depicted in different angles or whether they are actually different figures (i.e. mirrored figures). This visuo-spatial task requires to keep one object in one´s own working memory long enough to mentally rotate it in space and to check whether it matches the other figure or not. The MR performance of a person is indicated by the individual slope of the psychometric function that reflects the speed with which participants mentally rotate the figures in degrees per second. The non-rotational aspects of the task including working memory, matching, and response preparation are indicated by the individual intercept of the psychometric function [7].

Sex-related differences in cognitive domains are not only of academic interest, but appear to also play a role in different psychopathological states. It has been speculated that sex differences in typically developed individuals have implications on our understanding of autism spectrum disorders (ASD) that have been claimed within the EMB framework to exhibit an extreme form of a typical male cognitive profile [8]. More generally speaking, according to the extreme male brain hypothesis (EMB) [9], the typical female and male cognitive profiles reflect innate differences in the brains of males and females. The typical female cognitive profile has been classified as “empathising”, whereas the typical male cognitive profile is talented in “systemising”. Empathising refers to the ability to understand and feel other people’s feelings and emotions, whereas systemising is the skill of analysing and operating on systems [9]. The cognitive profile of individuals with ASD has been speculated to be characterised by an increased systemising and a decreased empathising performance as a continuous extension of the male cognitive profile. Such a “hypermale” cognitive profile is assumed to be possibly caused by the exposure to elevated levels of testosterone during ontogeny [8, 9]. The Intuitive Physics Test (IPT), a 20-item multiple choice questionnaire, was developed in order to assess the systemising ability in a standardised manner [10].

Interestingly, despite of the marked sex differences found in MR performance, the debate is not resolved, whether MR performance of individuals with ASD matches (or even exaggerates) male MR performance or not [2, 11, 12]. Challenging the EBM hypothesis of ASD we could show in one of our own studies that the cognitive profile exhibited by high-functioning children with ASD in the MR task as compared to TD children contrasted with sex-related differences between TD males and females. Employing exactly the same task ASD individuals outperformed TD males on the non-rotational aspects of the MR task [11]; note though the meta-analysis by Muth et al., 2014 [13] showing only scant overall evidence of superior MR performance in ASD), whereas, in contrast, TD neurotypical males outperformed TD females on the rotational, but not the non-rotational, aspects [3].

Despite this apparent contrast between the ASD-related MR advantage in the non-rotational components and the TD male MR advantage in its rotational components, it has been argued that sex differences might nevertheless reside in intercepts reflecting the non-rotational component rather than in slopes reflecting the rotational component [2]. Brosnan, Daggar, et al.’s (2010) [2] findings contrast with previous findings that found sex differences to reside in *slopes* of error rates [14] and *slopes* of inverse efficiency scores [3, 11] rather than intercepts. Given the discrepancy in findings, several methodological issues have been raised [14] and will be addressed in the current study as outlined below. A clarification of this inconsistency is obviously highly relevant for the EMB theory which would suggest males to outperform females on the same aspects of the MR on which individuals with ASD outperform typical male controls.

The current study aims to shed light on this inconsistency and clarify whether sex-related MR performance differences in TD individuals support or contrast this prediction of the EMB account. Thus, we sought to investigate whether sex differences in TD individuals reside in MR slopes or intercepts by using the original computerised three-dimensional MR version employed by Falter et al. [3, 11], allowing a valid comparison to performance in a sample of individuals with ASD [11] and we incorporate the following methodological suggestions.

First, we perform separate analyses of ‘same’ and ‘different’ trials in a MR task as judging two figures to be different takes more time than finding out that they are the same because in the former case the figure has to be rotated the full 360° to realise that it is at no point similar to the comparison figure, whereas in the latter case it is sufficient to rotate one figure to the same angle as the other figure to match them [14]. Second, as sex differences for accuracy are more pronounced at greater degrees of rotation, but not beyond 120 degrees, we now have extended the rotational angles to 120 degrees. In addition, our task uses a more fine-grained equidistant set of positions than that employed by [14] with increased sensitivity to test group differences in slopes. Third, we additionally measured IPT scores, handedness, and 2D:4D ratios [2], taking findings into account that left-handedness occurs more often in males than in females [15] and that more than 50% of ASD individuals are left-handed [16]. 2D:4D ratios (i.e. the ratio of second finger length to fourth finger length), which have been argued to indicate exposure to prenatal testosterone [17], were included for comparison reasons with the study by Brosnan, Daggar, et al. [2].Fourth, we analysed RT and accuracy scores separately [3] responding to the objection that the use of a bias-free inverse efficiency measure (calculated by dividing RT scores by accuracy scores) in our previous study [3] might have influenced results [2].

With attention to these methodological issues and by using the original MR task that had previously been used to test performance in an ASD group [11], we now for the first time (i) investigate conclusively whether sex-related differences reside in slopes or intercepts in TD males and females, (ii) compare results to previous findings using the same MR task in ASD, (iii) and explore whether IPT scores reflecting systemising, 2D:4D ratios, and handedness are related to MR performance.

## Method

### Participants

In the current study, 53 participants were recruited, however, two were excluded on the basis of performance (see Results section). Hence, 51 participants were included in the current analysis (25 females, 26 males) most of whom were psychology students from the University of Groningen, the Netherlands, who received study credits for their participation. All students gave their written informed consent prior to their inclusion in the study and ethics approval was obtained by the Ethical Committee Psychology of the University of Groningen prior to data collection. Female age ranged from 19 to 26 and male age ranged from 20 to 28 (see Table 1). All participants had normal or corrected-to-normal vision. The majority of the participants was right-handed (*n* = 21 females, *n* = 24 males), with only a minority being left-handed (*n* = 4 females, *n* = 2 males) as assessed using the Edinburgh Handedness Inventory [18].

**Table 1:** Demographic Data for Female and Male Participants.

		Minimum	Maximum	*Mean / Median* *	*SD / QD* *
Females (*N* = 25)					
	Age	19.30	26.10	21.40	11.99
	IPT	2	13	8.80	2.75
	2D:4D	.8900	1.0700	.9848	.0413
				
Males (*N* = 26)					
	Age	20.02	28.40	23.30	12.40
	IPT	7	17	11.96	2.55
	2D:4D	.9300	1.0600	.9791	.0327
				

*Note*. IPT = Intuitive Physics Test; *SD* = standard deviation; 2D:4D = second to fourth digit ratio.

* Median and QD in case of age data.

### Stimuli and Design

A computerised MR task (adapted from [6]; for a detailed description see [3]]) was conducted on a standard PC with a 19 inch screen using E-Prime 2.0 software [19]. Participants were simultaneously presented with two three-dimensional figures to the left and right of the centre of the screen and were instructed to judge as fast and accurately as possible whether the two figures were the same (i.e. rotated versions of each other) or different (i.e. mirror images of each other) by pressing one of two keys with the right and left index finger (‘m’ and ‘c’ respectively). The background of the screen was black and the figures were light blue. Each figure was made up of ten connected cubes. Seven different equidistant angles of rotation were used (0°, 20°, 40°, 60°, 80°, 100°, and 120°). Rotation angles and same/different (50% at each position) trials were presented in randomised (experiment-wise) order. The size of each figure was 3.8 degrees of visual angle vertically and horizontally; the distance between the midpoints of the figures was approximately 9.5 degrees All trials were preceded by a fixation cross with a duration of 500 ms. The MR task included 448 trials. In total, eight experimental blocks were conducted with 56 trials per block and a break in between blocks. Before the experimental blocks were initiated, a short practice session was performed in order to ensure that the participants understood the task requirements. Automatic auditory feedback was given when an incorrect answer occurred.

The IPT [10] which consisted of 20 multiple choice questions and for which participants were given a 10 minute time limit was administered. Higher scores on the IPT indicate better performance. A stop watch was used to measure the time. IPT scores for females and males are presented in Table 1. Left and right 2D:4D ratios were assessed using a digital calliper.

## Results

### Mental Rotation Variables

Accuracy scores (ACC) and reaction times (RT) of slopes and intercepts for same and different trials were analysed as dependent variables. Two participants were excluded from further analysis due to random response behaviour. Thus, the final sample size consisted of *N* = 51 (25 females, 26 males). RT and ACC data were not normally distributed and non-parametric tests were used throughout.

**Fig 1:**
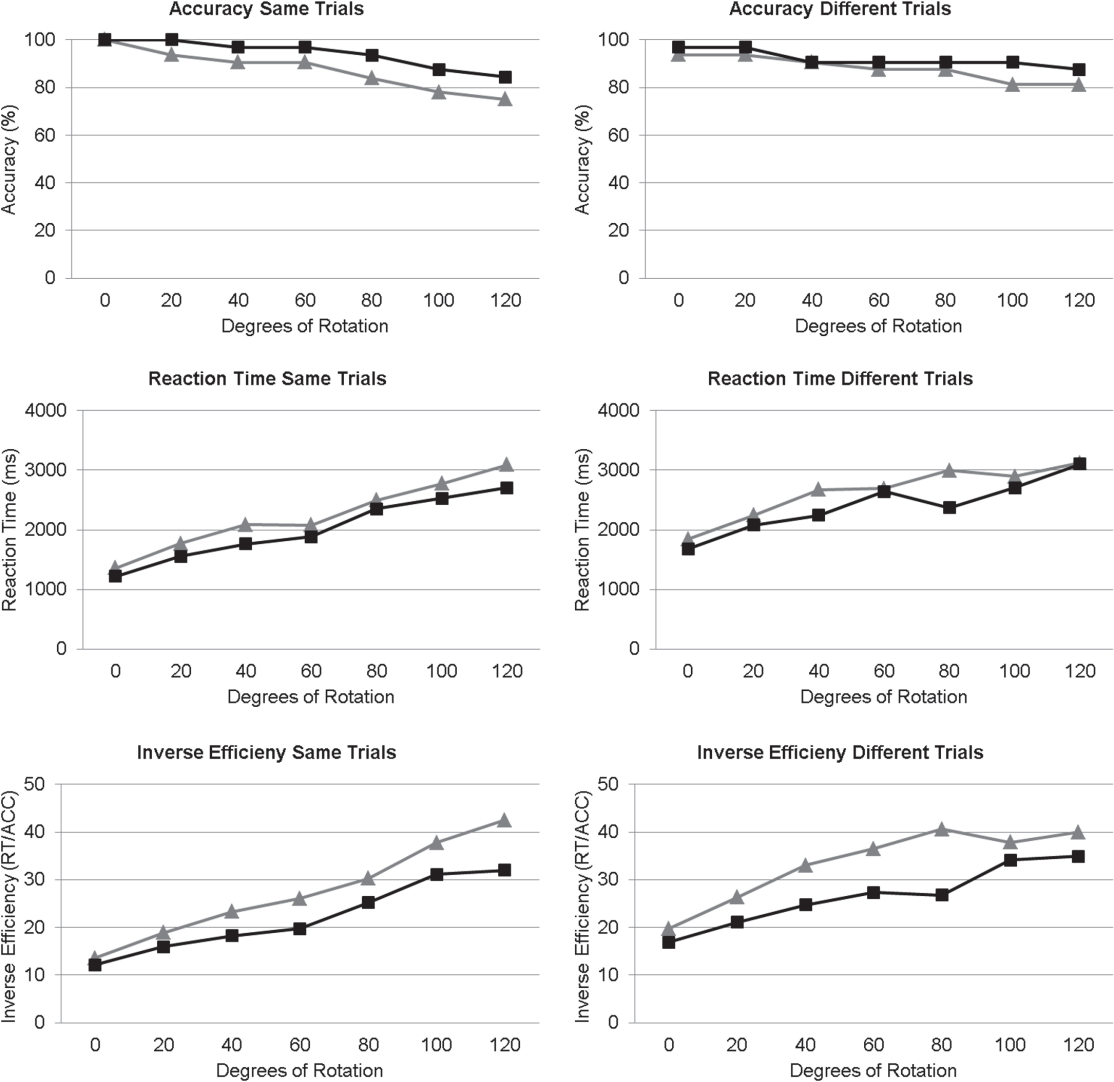
MR accuracy (in percentage), reaction times (in ms) and inverse efficiency scores (RT / accuracy) separately for same and different trials for female (grey triangles) and male (black squares) participants.

Mann-Whitney U-tests showed that ACC slopes were significantly shallower for male participants than for female participants for same trials, *U*(51) = 191.5, *p* = .012, *r* = -.35 (see Table 2), with a similar trend for different trials, *U*(51) = 224, *p* = .056, *r* = -.27, indicating that for greater angles of rotation males performed better on the task than females (see Fig 1). Concerning the non-rotational aspects of the MR (i.e. ACC intercepts) no significant differences between male and female performance were found, despite of a trend for different trials, *U*(51) = 229.5, *p* = .072, *r* = -.25.

**Table 2:** Overview of Female and Male Medians (QD) for Slopes and Intercepts of Same and Different Trials.

	Accuracy		Reaction time	
Condition	Male	Female	Male	Female
Slope, same	-.11 (-.01)	-.19 (.01)	10.35 (8.79)	11.92 (9.99)
Slope, different	-.06 (.01)	-.09 (<.01)	6.52 (6.60)	9.25 (9.12)
Intercept, same	100.66 (52.57)	99.32 (52.18)	1040.74 (723.75)	1306.63 (1004.79)
Intercept, different	96.99 (52.34)	92.30 (57.48)	1416.35 (1179.42)	1743.79 (1166.10)

*Note*. *QD* = Quartile Deviations.

In contrast to ACC, no significant differences between male and female participants for RT scores alone were observed (largest *U* = 258.5), showing that females and males were comparably fast in their MR performance (see Table 2).

### Composite scores

It has been argued [2] that different conclusions might be derived from analysis of bias-free composite scores as used by Falter et al. [3]. When submitting inverse efficiency scores (RT scores divided by accuracy scores) to the same analysis procedure, the conclusions are strikingly similar. Males and females differed significantly with respect to slopes of same, *U*(51) = 212, *p* = .024, *r* = -.32, and different trials, *U*(51) = 214.5, *p* = .027, *r* = -.31. In contrast, there were no significant sex differences on intercepts, but a trend for different trials, *U*(51) = 223, *p* = .055, *r* = -.27.

Likewise, it has been argued [2] that composite scores of same and different trials as analysed by Falter et al. [3] might lead to completely different conclusions. However, again, conclusions were similar when scores were analysed as composite measures. Thus, males significantly outperformed females, *U*(51) = 178, *p* = .006, *r* = -.39.

### Systemising

Males (*M* = 11.96) outperformed females (*M* = 8.80) significantly on the IPT, *t*(49) = -4.25, *p* < .001, Cohen’s *d* = 1.19. Spearman’s *ρ* was calculated between IPT and MR RT and ACC scores. There was a significant correlation of systemising with RT slopes of same (*ρ* = -.48, *p* = .014) and a trend of different (*ρ* = -.34, *p* = .094) trials. There were no significant correlations with intercepts (largest *ρ* = -.27). No significant correlations were found for females.

### Handedness

There was no sex difference of handedness, *t*(48) = -1.14, *p* = .260 and no correlation between handedness scores and MR performance (largest *ρ* = -.20).

### 2D:4D ratios

There was no difference in 2D:4D ratios, *t*(49) = .54, *p* = .595, Cohen’s *d* = .15, between males (*M* = .9791) and females (*M* = .9848). There was no correlation between 2D:4D ratios and MR performance (largest *ρ* = -.30) in males or females.

## Discussion

The current study aimed at clarifying previous inconsistent findings by testing to what extent MR performance differences in TD males and females reside in rotational components of the task (indicated by slopes) or non-rotational components (indicated by intercepts). In addition, the roles of IPT scores, handedness, and 2D:4D ratios were investigated and correlated with MR performance (see [2]). The three main results of this study, firstly, provide a detailed picture of sex-related differences in components and conditions of the MR task and, secondly, highlight that sex-related differences in TD individuals do not match the pattern of superior MR performance in ASD.

First, sex differences were found for ACC in the current study, not for RT, which is in accordance with previous research [2, 14] but in contrast to the difference in performance found for individuals with ASD and controls [11], who only showed an advantage for RT, but not for ACC scores.

Second, the current study showed a sex difference in slopes of TD individuals with shallower slopes in males than females. This finding replicates the result of sex differences in slopes reported by Falter et al. [3] and Brosnan, Walker, et al. [14] and corroborates the difference in performance pattern found for TD males versus females on the one hand, and individuals with ASD versus typically developing controls on the other hand [11, 12].

**Fig 2:**
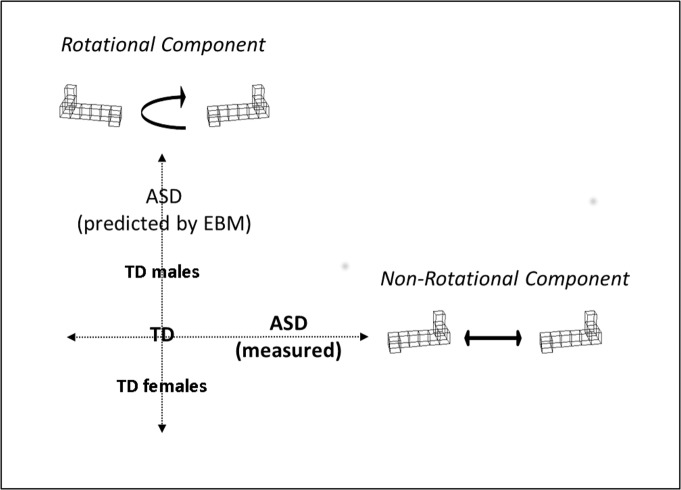
Typical examples of 3D figures used in the current Mental Rotation task. Cognitive processes related to task performance can be divided into independent rotational and non-rotational components. Typical sex differences were observed within the rotational dimension, whereas variation related to diagnosis was observed within the non-rotational dimension.

Third, in contrast to Brosnan, Daggar, et al. [2], but in line with Falter et al. [3] and Brosnan, Walker, et al. [14], who did not find an overall sex difference for error rate intercepts, there was no sex difference found for intercepts in the current study (neither ACC nor RT). Prior research has shown that individuals with ASD outperformed TD individuals only on RT intercepts [11]. However, a recent study revealed that ASD participants had slower processing (i.e. higher intercepts) compared to typically developing controls [20]. While we interpreted our previous results in the light of a local feature-based strategy [11], the results by Pearson et al. [20] were interpreted in the context of a configural strategy. In addition, Pearson et al. [20] used pictures of bodies or cars as stimuli, while we used geometric figures. These findings corroborate the idea of a different cognitive profile for MR in ASD that contrasts the different cognitive TD sex-related profiles, which contradict the EMB theory (see Fig 2).

It was argued by Brosnan, Daggar et al. [2] that the use of composite scores might influence the results and lead to the formulation of different conclusions. However, the current study demonstrates that for composite scores of RT and ACC (i.e. bias-free inverse efficiency scores derived from dividing RT by ACC), the conclusions are strikingly similar as compared to considering RT and ACC scores separately. In both scenarios, ACC slopes have found to be shallower for males, indicating that they outperform females on the MR components of the task. The same holds true in the case of same and different trails. The use of a bias-free composite score leads to the same conclusion as when analysing same and different trials separately. In both cases, males performed better than their female counterparts. Admittedly, when analysing same and different trials separately as suggested by Brosnan, Walker, et al. [14], a trend was found for lower ACC intercepts of different trials in males showing that there might be a slight advantage also in non-rotational aspects of the task for TD males over females, although not significant and only confined to different trials. Interestingly though, this trend is in contrast to the trend found by Brosnan, Walker, et al. [14] of males having higher RT intercepts reflecting a worse non-rotational performance than females in both same and different trials. Thus, given that these non-significant trends in both studies run in opposing directions, it is reasonable to suggest that there is no statistically significant sex-related difference for intercepts in MR.

Overall, these three findings strongly point towards different patterns of performance found in individuals with ASD versus TD controls [11] and the pattern of sex-related differences in TD individuals found in the current as well as previous studies [3, 14], even though several methodological suggestions were taken into account such as splitting analysis in ‘same’ and ‘different’ trials, extending the range of rotation angles, and analysing RT and ACC scores separately (as requested by Brosnan, Walker et al. [14]). Importantly, while Brosnan, Walker et al. [14] tested MR performance in a TD group only, with limited implications for the cognitive profile in ASD, the current findings provide strong support for a contrast of ASD-related differences on the one hand and sex-related differences within the TD group on the other hand given that the version of MR task used in the current study was the same as that tested previously in males and females [3] and in individuals with ASD [11].

What might therefore at least partly explain the inconsistency of the current findings to those by Brosnan, Daggar, et al. [2] is the use of a different MR design in the latter study. For instance, in the current study we used 0, 20, 40, 60, 80, 100, and 120 degrees of rotation, whereas Brosnan, Daggar, et al. [2] only tested 0, 40, 80, 120, and 180 degrees. Not only did the current task version include a larger variety of angular disparities but also relatively small and equidistant increases in degrees of rotation. The slope measure in the current study might therefore be more sensitive to pick up subtle sex-related differences than the slope measure used by Brosnan, Daggar et al. [2].

Another factor providing a possible explanation for the different findings is the age of participants. In a recent study conducted by Jansen and Heil [21] the effect of age on MR performance was investigated systematically: performance in general was found to decrease with age. The sex effect was found among all age groups, however, the size of the effect decreased with age. Rotational and non-rotational components were not analysed separately, though the authors reported no gender difference for the 0° trials [21] which, as no rotation is necessary, indicates that there are no sex-related differences in the non-rotational aspects of the task. This finding implies that the decrease in performance with increasing age resides in the rotational aspects of the MR. In the described study, three age groups (20–30; 40–50; 60–70) were employed whereas in our current study, age ranged from 19 to 28 with a mean around 22. The mean age of the participants in the study conducted by Brosnan, Daggar, et al. [2] was 30. Both studies would therefore best fit with the first age group created by Jansen and Heil [21]. However, the average age difference of the participants in the study performed by Brosnan, Daggar, et al. [2] amounts to almost ten years more compared to the participants in the current study. A similar age difference can be seen in Brosnan, Walker, et al. [14], who also found sex differences to reside in rotational aspects of MR error rates. A study targeting the analysis of rotational and non-rotational components of MR across age groups might be useful to obtain conclusive results as to whether age might be a reason for inconsistency of previous findings.

Besides the location of sex-related differences in the MR task components, several predictions can be deducted from EMB theory with respect to systemising in relation to MR (see Brosnan, Daggar, et al. [2]). According to EBM, (i) TD males should outperform females in a test of systemising, and, importantly, (ii) systemising should correlate with those MR task components at which ASD individuals excel (i.e. RT intercepts). Male participants in the current study did outperform females on systemising which is in accordance with EMB theory (see also Brosnan, Daggar, et al. [2]). In addition, systemising did correlate with MR task performance only for males, but not on task aspects at which ASD individuals excel (i.e. RT intercepts, see Falter et al. [11]). Instead, systemising correlated with RT slopes, not on intercepts or ACC scores. Surprisingly, in the study by Brosnan, Daggar, et al. [2] systemising correlated with ACC intercepts. However, again different MR task versions might have contributed to these differences in findings. Importantly, given that individuals with ASD excel at RT intercepts, both the current study as well as the study by Brosnan, Daggar, et al. [2] show different patterns of correlations than expected by EMB theory. In addition, given the same MR task version previously tested in individuals with ASD [11] and the current study, it is safe to say that individuals with ASD excel at different aspects of the MR task than those task aspects related to systemising (and typical male task superiority) (see Fig 2).

For reasons of comparison, we have also tested, whether MR performance correlates with handedness and 2D:4D ratios (see Brosnan, Daggar, et al. [2]). There was no sex difference and no correlation with MR performance concerning both 2D:4D ratios, corroborating previous results by Falter et al. [3], and handedness. Brosnan, Daggar, et al. [2] also reported no significant relationships between handedness and MR performance on rotational as well as non-rotational aspects. The null findings indicate that there are no structural differences concerning the lateralisation of spatial abilities. However, McGee [22] found an interaction effect between gender and handedness on MR ability and Reio, Czarnolewski, and Eliot [23] related left-handedness to spatial ability and MR. The findings of the present study with respect to handedness have to be interpreted with great caution though because of unequal sample sizes of left- and right-handers as opposed to the other studies. Given the findings by Dane and Balci [16], who have shown that rates of left-handedness are quite high among autistics and that lateralisation differs from typically developing individuals, handedness might be a factor interesting to take into account in future research studies, provided a more similar group size.

In conclusion, the current findings have implications for EMB theory in that the theory would predict that TD males outperform females on the same aspects of the MR task on which individuals with ASD outperform TD controls. However, the MR performance difference between ASD and TD individuals was found to lie within the RT intercepts, whereas there was no group difference concerning slopes [11], in contrast to typical males outperforming typical females on MR slopes in the current study. The current study therefore replicates the findings reported by Falter et al. [3] and corroborates the argumentation that a difference exists between typical male performance in MR and performance patterns seen in individuals with ASD [11, 12]. Thus, there is now converging evidence using the same computerised MR task in three studies testing TD males and females as well as individuals with ASD, taking several methodological issues, a systemising measure, and indices of prenatal testosterone and laterality into account, that the pattern of autism-control difference is not the same as the cognitive sex differences in MR performance. Therefore, the current findings bear relevant implications for the scientific and clinical field of autism by showing that autistic people do not have an extreme version of a male cognitive profile as proposed by the EMB theory.
